# Food and Beverage Marketing in Schools: A Review of the Evidence

**DOI:** 10.3390/ijerph14091054

**Published:** 2017-09-12

**Authors:** Cayley E. Velazquez, Jennifer L. Black, Monique Potvin Kent

**Affiliations:** 1 Faculty of Land and Food Systems, Food Nutrition and Health, University of British Columbia, 2205 East Mall, Vancouver, BC V6T 1Z4, Canada; j.black@ubc.ca; 2 Faculty of Medicine, School of Epidemiology and Public Health, University of Ottawa, 600 Peter Morand Crescent, Ottawa, ON K1G 5Z3, Canada; mpotvink@uottawa.ca

**Keywords:** advertising as topic, marketing, food, beverage, food advertising, food marketing, youth, school

## Abstract

Despite growing interest from government agencies, non-governmental organizations and school boards in restricting or regulating unhealthy food and beverage marketing to children, limited research has examined the emerging knowledge base regarding school-based food and beverage marketing in high-income countries. This review examined current approaches for measuring school food and beverage marketing practices, and evidence regarding the extent of exposure and hypothesized associations with children’s diet-related outcomes. Five databases (MEDLINE, Web of Science, CINAHL, Embase, and PsycINFO) and six grey literature sources were searched for papers that explicitly examined school-based food and beverage marketing policies or practices. Twenty-seven papers, across four high-income countries including Canada (*n* = 2), Ireland (*n* = 1), Poland (*n* = 1) and United States (*n* = 23) were identified and reviewed. Results showed that three main methodological approaches have been used: direct observation, self-report surveys, and in-person/telephone interviews, but few studies reported on the validity or reliability of measures. Findings suggest that students in the U.S. are commonly exposed to a broad array of food and beverage marketing approaches including direct and indirect advertising, although the extent of exposure varies widely across studies. More pervasive marketing exposure was found among secondary or high schools compared with elementary/middle schools and among schools with lower compared with higher socio-economic status. Three of five studies examining diet-related outcomes found that exposure to school-based food and beverage marketing was associated with food purchasing or consumption, particularly for minimally nutritious items. There remains a need for a core set of standard and universal measures that are sufficiently rigorous and comprehensive to assess the totality of school food and beverage marketing practices that can be used to compare exposure between study contexts and over time. Future research should examine the validity of school food and beverage marketing assessments and the impacts of exposure (and emerging policies that reduce exposure) on children’s purchasing and diet-related knowledge, attitudes and behaviors in school settings.

## 1. Introduction

Improving the eating habits and nutritional health of children and adolescents is a priority area for public health efforts in North America and Europe [1,2], particularly because the prevalence of adverse nutrition-related outcomes, including type 2 diabetes, and obesity, remains high [3]. Childhood and adolescence is frequently recognized as a critical time for the development of healthy eating habits [4,5,6]. Yet international findings indicate that most school-age youth in high-income countries do not meet dietary recommendations [7,8,9,10,11,12], and instead consume diets that are too high in total energy, saturated fat, sugar, and sodium, and too low in fruits, vegetables, whole grains and other key nutrients such as calcium, vitamin D, and fiber [7,8,13]. Moreover, diet quality may decline as children become older and move into secondary school [14], where intake of fruits, vegetables, and milk decrease and consumption of sugar sweetened beverages (SSBs) increase [15,16].

The determinants of dietary intake are complex, and food choices are influenced by a multitude of factors. Approaches to improving the dietary behaviors of children and adolescents frequently focus at the individual- or social environmental-levels [17], however recent evidence indicates that the physical environment, which includes the food environment, within communities may shape children’s dietary practices above and beyond these factors [18,19,20,21]. The food environment has been conceptualized by Glanz et al. as including four aspects: (1) the community nutrition environment (e.g., type and location of food outlets); (2) the consumer nutrition environment (e.g., availability of healthy food options); (3) the organizational nutrition environment (e.g., food access in settings such as schools) and; (4) the information environment (e.g., food marketing and advertising) [22]. Both individual perceptions and physical characteristics of the food environment can impact food-related decisions, influencing the type and amount of food and beverages consumed, and thus overall dietary quality and health outcomes [19,23]. 

Children and adolescents spend many of their waking hours at school where they consume approximately one-third of their daily energy intake [24,25]. Therefore, understanding how school food environments impact food choices is particularly important and a potentially salient component of emerging public health approaches to improving youth dietary outcomes. School food environment assessments are increasingly common in the academic literature [26,27,28,29], yet most have overlooked the ‘information environment’ that lies within these settings. Evidence suggests that food and beverage marketing impacts children’s food preferences, requests, choices, and health-related outcomes including overweight and obesity [30], and that exposure differs by race/ethnicity and income [31]; however, much of this work has focused on exposure channels (e.g., television) rather than settings (e.g., schools) [30]. In 2009, in the United States (USA) alone, nearly $150 million was spent by food and beverage companies to market their products in schools [32] since young people constitute a captive audience with tremendous buying power [33]. Commonly reported school food and beverage marketing activities include: (1) direct advertising (e.g., advertisements placed around campus on scoreboards, billboards, or posters); (2) indirect advertising (e.g., corporate sponsored curricula, coupons, or contests); (3) product sales (e.g., exclusive soft drink contracts, cash rebate programs) and; (4) market research (e.g., surveys, panels, or taste-tests) [34,35,36]. 

Despite interest from a variety of stakeholder groups including government agencies, non-governmental organizations and school boards in regulating food and beverage marketing to children in several countries [37,38,39], key questions remain. To date, no comprehensive review has synthesized the current knowledge base, assessed the methodological quality of current studies, or identified key gaps in the literature related to researching or evaluating school food and beverage marketing practices. To address current gaps in knowledge, the objectives of this study were to: (1) identify and summarize approaches for assessing the food and beverage marketing environment in schools; (2) examine current evidence regarding the extent to which students in developed countries are exposed to food and beverage marketing at school; (3) determine whether exposure differs by student- and/or school-level characteristics; (4) assess whether exposure is associated with students’ diet-related outcomes; and (5) identify information gaps and future research directions needed to inform emerging policies and practices designed to reduce the deleterious impacts of school-based food and beverage marketing. 

## 2. Materials and Methods

**Figure 1:**
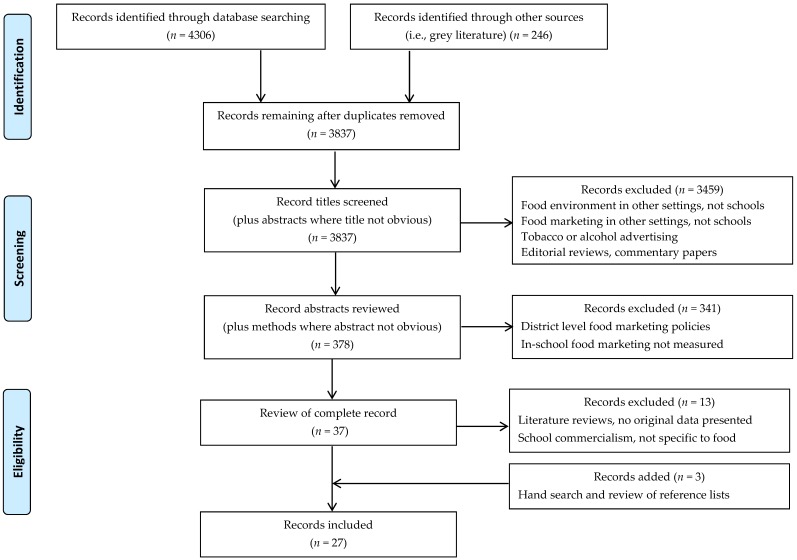
Flow diagram of records identified through review.

We searched five academic databases (MEDLINE, Web of Science, CINAHL, Embase, and PsycINFO) and six relevant grey literature sources (i.e., the Grey Literature Report, the World Health Organization Library Database, the World Advertising Research Center website, the Food Marketing Workgroup website, the National Cancer Institute instrument database, and the Rudd Center for Food Policy and Obesity website) for documents published prior to 2016. The search strategy included a combination of medical subject heading (MeSH) searches and free-text searches using commonly used terms (see Figure 1). The initial search strategy was developed in MEDLINE, and was iteratively refined and adapted for the remaining databases. Database and grey literature source searches were completed between 14 and 31 July 2015.

Marketing (including to children at schools) occurs by integrating several tools: product, price, place, and promotion, collectively known as the ‘marketing mix’ [40]. The primary focus of this review was the study of ‘promotion’ described as the persuasive communication techniques used to relay information to the consumer, of which advertising is a large part. More specifically, this review was guided by the 2012 World Health Organization report on the marketing of foods and non-alcoholic beverages to children [36], in which commercial activities at schools are grouped into one of four categories: direct advertising, indirect advertising, product sales, and market research. The main search terms were therefore organized as follows: (1) marketing (e.g., advertising, sponsorship, coupons), (2) food (e.g., fast food, sugar-sweetened beverages, candy), and (3) schools (e.g., elementary, middle, high), with pre-determined limits of English language publications using only human subjects. To begin, reference titles (and abstracts where the title was not obvious) were reviewed. References were retained if they included a general focus on the school food environment (first round of review), followed by a review of the full abstracts. References were retained if they included a specific focus on food or beverage marketing policies or practices in schools (second round of review). Studies were excluded if the setting was anything other than schools (e.g., homes, daycares). Studies solely discussing the availability or sale of competitive foods were excluded because they fell outside the ‘marketing mix’ category (i.e., promotion) of interest, as were studies that did not specifically describe school-level marketing exposures (e.g., studies that focused on school district or state-level policies only). A hand search was conducted and reference lists were further screened to identify additional studies. 

A data extraction form was developed and key information was pulled from each article. Information pertaining to methods of data collection and administration time, instrument development and psychometric properties, and key measured marketing variables were extracted to summarize the approaches used to measure food and beverage marketing practices in school settings (Table S1). Additionally, current evidence about school food and beverage marketing exposure, differences in exposure by student- and/or school-level characteristics, and associations with diet-related outcomes were also extracted from each article (Table S2). This review was informed by the Preferred Reporting Items for Systematic Reviews and Meta-Analyses (PRISMA) guidelines [41].

## 3. Results

Table S1 summarizes the 27 cross-sectional studies (*n* = 20 unique data sources) from high-income countries that met the inclusion criteria. More than half (*n* = 15) of the studies were published between 2010–2015, and only two [42,43] before 2005. The majority of studies (*n* = 23, *n* = 16 unique) were conducted in the US, with the remainder drawn from Canada (*n* = 2), Ireland (*n* = 1), or Poland (*n* = 1). Ten studies (8 unique) sampled only secondary schools (e.g., grades 9–12), two studies included only elementary schools (e.g., kindergarten-grade 7), and one study included only middle schools (e.g., grades 6–8). The remaining studies (*n* = 14, *n* = 9 unique) were comprised of some combination of elementary, middle or secondary schools. 

### 3.1. Overview of Approaches for Assessing School Food and Beverage Marketing

Three methodological approaches have been used to measure food and beverage marketing exposure within schools: direct observations, self-report surveys, and in-person or telephone interviews with school administrators or staff. Twenty-three studies (*n* = 16 unique) used one method of assessment and four studies used two methods in combination; eight studies directly measured food and beverage marketing in schools and 23 (*n* = 16 unique) studies used indirect observation to measure individuals’ (e.g., administrators’) perceptions of the school food and beverage marketing environment. 

#### 3.1.1. Direct Observations

Direct observations were conducted in 8 studies [44,45,46,47,48,49,50,51] completed by either: (1) trained research staff/project coordinators (*n* = 6); (2) university or high school students (*n* = 2) and/or; (3) school nurses (*n* = 1). The reported time to administer various tools ranged between 1 and 3 h per school. The California Project Lean (CPL) School Food and Beverage Marketing Assessment Tool (FBMS) [35] was the only instrument used in multiple studies as it was developed for one study [46] and adapted/used by three other studies [45,47,50]. The remaining tools were adapted from studies assessing food and beverage marketing in other channels (e.g., television) or newly created. Some form of instrument-, field-, or pilot-testing (four studies) and/or intra- or inter-rater reliability (four studies) was cited as having been completed among direct observation studies. Where noted, observational tools or coding protocols exhibited moderate or strong inter-rater reliability with Spearman correlations ranging from 0.56 to 0.72 (where moderate is 0.40–0.59 and strong is 0.60–0.79) [50] and Cohen’s Kappa ranging from 0.78 to 0.95 (where substantial and near perfect agreement are 0.61–0.80 and 0.81–1.0, respectively) [47,48,51]. 

All studies using observational methods assessed some form of direct advertising (e.g., ads in school buildings). Typically, these studies captured the number, type, and locations of food and beverage advertisements, and the kind and relative healthfulness of products promoted compared to national standards such as USDA foods of minimal nutritional value [52] or regional guidelines [53]. Some studies further assessed presence of indirect advertising, product sales, or market research. Compliance with laws or policies restricting food and beverage marketing in schools was examined in two studies [50,51]. Studies using observational methods tended to capture more specific details, such as number of food and beverage marketing instances and types of products promoted within schools, which allowed for a greater understanding of the frequency and nutritional content of products featured in the marketing.

#### 3.1.2. Self-Report Surveys or Questionnaires

Nineteen studies [24,42,43,49,54,55,56,57,58,59,60,61,62,63,64,65,66,67,68] (drawing from 12 unique research projects) used self-report surveys, where respondents were asked to independently answer questions such as their perceptions of the school food and beverage marketing environment. Eighteen studies (using 11 unique surveys) were completed by school personnel, typically a principal or food service director, and one study was a web-based questionnaire completed by students. Time to complete the surveys was reported to be between 15 and 45 min although many also included measures unrelated to school food marketing. Several studies using self-report surveys used the same or very similar questions, for example, “Does your district or school have an existing exclusive beverage contract in place?” to obtain information about the frequency of food and beverage marketing and related policies. Pilot-testing was reported as completed for seven out of 19 studies (four of which were unique), however, just three studies (all appeared to draw on the same data set) reported undergoing psychometric testing, where phone interviews were conducted to validate key variables of interest. No studies mentioned the type of validity assessed or key findings from validation approaches.

All studies using self-report survey methods assessed perceptions of food and beverage marketing in schools, and often assessed direct advertising related to the type (e.g., posters, supplies, curricula) and location (e.g., cafeteria, hallway, classroom) of marketing. Ten studies (six unique) examined product sales in the form of an exclusive beverage contract and six studies (four unique) examined whether schools had a policy restricting food and beverage marketing. Still, other studies aimed to understand the prevalence of indirect marketing such as textbook covers, coupons, or sponsorship of school events. Studies using self-report surveys commonly focused on the presence/absence of a particular form of food and beverage marketing in schools.

#### 3.1.3. Interviews

Four studies [45,46,50,69] used in-person or telephone interviews, typically in combination with direct observation methods. Interviews were usually conducted with school administrators or food service directors, either by trained research staff/project coordinators or in one study, dietetic interns. Interviews reportedly took between 10 and 40 min to complete. The interview questions were not reported as having been adapted from other sources. 

Studies using in-person or telephone interview methods were designed to capture aspects of the food and beverage marketing environment that are not always obvious during school walk-throughs. For instance, this assessment method frequently focused on policies pertaining to food and beverage marketing within schools, as well as whether exclusive beverage contracts, corporate sponsored fundraising, scholarships, or sponsorship of school events, and/or indirect advertising such as the use of textbook covers, coupons, or equipment and supplies were allowed. 

#### 3.1.4. Strengths and Limitations of Assessment Approaches

Observational techniques offer an opportunity to directly assess food and beverage marketing and often provide a greater level of detail about the total number of advertisements and specific products promoted than other approaches. Observations may minimize bias compared to subjective reports, particularly when photographs are taken because they can aid in coding activities and do not rely on respondents’ memory or personal knowledge. However, this method is time consuming and generally takes a greater number of resources to complete, limiting study scope and sample size. Alternatively, self-report surveys and interviews can be used when perceptions are of interest, resources are limited, or if the target sample is large and difficult to reach in person. Self-report approaches are subject to bias (e.g., social desirability, where respondents answer in a way that portrays their school in a favorable manner) and reporting error (e.g., under- or over-reporting) given that they rely on accounts from school administrators or staff. Since respondents may not be aware of all of the various types of food and beverage marketing practices within their school, the full scope of activities may be missed. Still, this approach offers the opportunity to inquire about less visible forms of marketing such as scholarship programs or fundraising efforts that cannot be directly observed.

### 3.2. Exposure to Food and Beverage Marketing in Schools

Table S2 summarizes the studies that have described students’ food and beverage marketing exposure in schools in developed countries published prior to 2016. Generally, exposure was described within three main categories: (1) direct advertising; (2) indirect advertising and; (3) product sales. Findings pertaining to the fourth category (e.g., market research) [34,35,36], were not commonly reported and therefore not included here. Two studies formally assessed compliance with food marketing laws or policies according to specific nutrition guidelines, while other studies rated the relative healthfulness of the items advertised in schools. 

#### 3.2.1. Direct Advertising

Two nationally representative studies using self-report survey approaches from the US [66,69] described relatively low prevalence (<10% of schools sampled) of direct advertising by way of appropriation of space (e.g., the posting of a brand or logo, for example on scoreboards). These findings contradict several studies which yielded substantially higher estimates of the frequency and prevalence of marketing activities. For example, in other regional or state-representative studies, the most common forms of direct advertising were via posters or signs found in 83–90% of schools sampled [45,46] and vending machines found in 62–100% of schools [44,64]. Food and beverage coolers or display cases, as well as school yearbooks were also cited as advertising corporate logos or brand names [46,50]. Advertisements were frequently reported in locations such as cafeterias, hallways, and gymnasiums/playing fields [48,51,64]. Advertisements in other locations, for instance school newspapers, public-address systems, or buses, were reported less often [46,64]. 

#### 3.2.2. Indirect Advertising

Indirect advertising was assessed in slightly less than half (*n* = 11) [42,43,45,46,50,56,57,58,63,66,69] of the studies included here. Four studies [35,45,58,69] reported on the use of educational materials or curricula created by food companies, where use ranged from 2% [69] to 26% [35] of schools. Sponsorship of school events was reported in seven studies [45,50,56,57,58,66,69]. Although one study [45] reported that less than 5% of their randomly selected sample of Maryland (USA) schools marketed foods via sponsorship, other studies found the proportion to be higher. Using nationally representative samples, sponsorship of programs or activities was reported in 11% of US elementary, middle, and high schools [69] and 21% of US high schools [66]. Sponsorship of competitions or events were reported in two studies, occurring in 40% of Irish schools [58] and 45% of a sample of Maine (USA) schools [50]. Only seven studies examined the use of coupons. French et al. [43] found that 22% of the 336 respondents from a state-wide census survey of Minnesota (USA) high school principals allowed food/beverage coupons to be distributed to students, and, while Terry-McElrath and colleagues [66] found that food coupons were the most frequent type of commercialism in elementary schools (e.g., 64% of students were exposed to food coupons), they were less prevalent in middle and high school (e.g., just 5% and 6% of students, respectively, received food coupons).

#### 3.2.3. Product Sales

Products sales, primarily exclusive beverage contracts, were measured in 15 studies [24,42,43,45,46,50,55,56,57,64,65,66,67,68,69]. Molnar [69] reported that exclusive agreements were present in 21% of schools sampled across the USA, however other studies reported that 49% [64], 55% [55], 70% [66], and nearly 80% [43] of sampled schools had a contract with either a food or beverage company. Food-based fundraising with branded products was also reported. One study [46] found that all 20 public high schools in their California (USA) sample had used branded food and beverage products in fundraisers, where others found that these activities were present in 36% [69], 50% [45], and 70% [50] of schools. As noted in work from the Center for Science in the Public Interest (CSPI) [45], fundraisers typically were for candy, baked goods, soda, or fast food or other restaurant food; items that tend to be energy dense and nutrient poor and discouraged for consumption given their misalignment with current nutrition guidelines. 

#### 3.2.4. Policies and Guidelines

Presence (or absence) of policies or guidelines regulating food and beverage marketing in schools were examined in 8 studies [45,46,58,59,60,62,63]. In a census survey of Irish schools, Kelly et al. [58] reported that <10% of sampled schools had a policy regarding commercial sponsorship from food companies. CSPI [45] found that only half of the 36 schools sampled had a verbal/written policy specifically addressing food advertising. Similarly, in a random sample of Minnesota (USA) middle/high school principals, Larson and colleagues [59] found the proportion of schools with policies that banned advertising on school grounds or buildings to be 46% and 61%, respectively. Additionally, Phillips et al. [63] indicated that slightly less than half (43%) of their Arkansas (USA) sample (*n* = 832 schools) reported a policy prohibiting commercial food advertising on campus.

Two studies [50,51] examined whether items promoted in schools complied with laws or policies restricting school food and beverage marketing. Polascek and colleagues [50] examined compliance with a state of Maine (USA) law that limits the marketing of foods of minimal nutritional value (FMNV) based on USDA standards for FMNV 52 in public schools (the USDA FMNV standards in schools were replaced with the Smart Snacks in School standards beginning in school year 2014–2015). The authors found that marketing of non-compliant items, for example Coca-Cola or Pepsi, was present namely on vending machines and scoreboards in 85% of their randomly selected sample of 20 schools. Overall, an average of 12 instances of non-compliant marketing was found per school. Similarly, Velazquez et al. [51] examined school-level compliance with a district-wide policy prohibiting commercial products from being advertised in schools in Vancouver (Canada), unless approved as having explicit educational value. Promotions for commercial items were identified in ~45% of schools, and comprised nearly 1/3 of all promotions. Additionally, more than half (55%) of the 23 schools sampled had promotions for items prohibited for sale by provincial (British Columbia) school nutrition guidelines [51].

#### 3.2.5. Relative Healthfulness of Products Marketed

While beverage vending machines in samples of Canadian and American schools frequently depicted advertisements for branded bottled water (e.g., Dasani or Aquafina) [44,50,51], advertisements featuring SSBs (e.g., Snapple, Powerade, Gatorade) were also common [44,50,51]. Other studies found nutrition education messages on 13% [51], 30% [48], and 49% [46] of advertisements; yet many studies indicated that marketing promoted unhealthy products [45,46,47,48,49,51]. For example, CSPI [45] found that 58% of food and beverage marketing posters were for restaurants, prepared foods, and soft drinks. Similarly, Craypo [46] reported that nearly 65% of vending machine advertisements were for SSBs, where Findholt [47] and Mazur [49] found that ~60% of messages in classrooms and school stores, respectively, were for ‘unhealthy’ products. 

### 3.3. Differences in Food and Beverage Marketing Exposure by Student- and/or School-Level Characteristics

#### 3.3.1. School Type

Three studies [51,57,66] found that advertisements were significantly more prevalent among secondary schools than middle/elementary schools, as was food company sponsorship [56]. Another study [45] found that the amount of advertising varied by grade level (e.g., branded curricula is more prevalent in elementary schools, whereas vending machine marketing is more frequent in middle/high school) and one study found that having a policy prohibiting commercial food advertising on campus was more likely among elementary schools than high schools [63]. Moreover, in a nationally representative sample of 287 US schools, Briefel et al. [24] found that the presence of an exclusive beverage contract was significantly more common in high schools than elementary schools; other studies echoed these findings [55,56,57,67]. One Polish study [49] found no significant differences in advertising by school type and another Minnesota (USA) study found that the mean number of locations where schools banned advertising did not differ by school type [59]. 

#### 3.3.2. Socio-Economic Status

Three studies [48,57,66] found significant associations between advertising exposure and SES. Drawing from a nationally representative sample of US schools and students, Johnston et al, [57] found that exposure to soft drink advertising differed by SES (using self-reported parental education as a proxy), where a greater percentage of students in low-SES high schools were exposed to such advertising compared with students in high-SES high schools (29% versus 13% respectively, *p* < 0.001). Similarly, Terry-McElrath et al. [66] found that an exclusive beverage contract in middle/high schools was significantly more likely in schools characterized as mid- or low-SES schools, defined based on the proportion of students eligible for free/reduced price lunches (15–39% and ≥40% respectively); yet Finkelstein et al. [55] found no statistically significant differences in the presence of an exclusive beverage contract between schools where ≤50% students qualified for free/reduced lunch (considered the proxy for higher-SES schools) compared to schools where >50% of children qualified for free/reduced lunch. One study of middle schools in Texas (USA) [48] found a significant association in the opposite direction, such that the mean number of advertisements was significantly higher for more affluent schools than more economically disadvantaged schools (≥60% of students qualified for free/reduced price lunch) (*p* < 0.001). Larson et al. [59] found that the mean number of locations where schools banned advertising did not differ by school SES.

#### 3.3.3. Racial/Ethnic Background

Johnston et al. [57] found some differences in advertising exposure according to the racial/ethnic makeup of students in sampled schools. For instance, the percentage of students in middle schools allowing soft drink bottlers to advertise was higher among schools with higher proportions of students characterized as primarily White or Black compared with predominantly Hispanic students. Terry-McElrath and colleagues [66] found differences in exclusive beverage contracts by student race/ethnicity for both middle and high schools (where exposure was significantly higher for students in schools with predominately White versus Black students); however, Finkelstein et al. [55] reported no statistically significant differences. Students attending high schools with predominately White students compared to those with mainly Black or Hispanic students were also significantly more likely to be exposed to school event sponsorships [66]. Larson [59] found the mean number of locations where schools banned advertising did not differ by ethnic minority enrolment levels, but Nanney et al. [62] did. Latimer [48] reported the mean number of advertisements as significantly lower among schools with a higher percent minority students (*p* < 0.001). 

#### 3.3.4. Geographic Location

Adachi-Mejia et al. [44] examined whether exposure to advertising differed by geographic location (e.g., urban, town and rural) among 26 high schools in New Hampshire (USA) and Vermont (USA), but found no statistically significant differences. Similarly, Larson [59] reported no statistically significant differences in the mean number of locations where schools banned advertising by geographic location, however, Nanney et colleagues [62] indicated that the mean number of banned locations was higher for urban and suburban schools that town/rural schools. Similarly, in a state-wide random sample of Minnesota (USA) middle/high school principals, Caspi et al. [54] indicated that both city and suburban schools were significantly more likely than rural/town schools to ban advertisements for low nutrient energy dense foods on school grounds. 

### 3.4. Associations between Exposure to Food and Beverage Marketing and Diet-Related Outcomes

#### 3.4.1. Food Purchasing and Consumption

There is some cross-sectional evidence (3 of 5 studies) that exposure to food marketing at school may shape food purchasing and consumption, particularly for minimally nutritious food items. For example, Mazur et al. [49] found that advertising a specific food on school store windows was associated with purchase of that food (*p* < 0.001), and Minaker et al. [61] found that perceived presence of snack or beverage logos at school was associated with a higher frequency of students purchasing food or beverages from vending machines (*p* < 0.0001). Additionally, Minaker et al. [61] found that students who reported the presence of snack logos in their schools were 1.3 times more likely to consume both salty snacks and candy than students who reported no logos (*p* < 0.0001 for both), but the reported presence of beverage logos in schools was not significantly associated with soft drink consumption (*p* = 0.06). Findings from Briefel and colleagues [24] indicated that energy intake from SSBs was significantly higher among students attending schools with an exclusive beverage contract. Larson et al. [59] found that the total number of locations where a school banned advertising was unrelated to school-level mean intakes of fruit, vegetables, and SSBs.

#### 3.4.2. Other Diet-Related Outcomes

The extent of soft drink advertisements in schools (e.g., schools with a higher number of locations where soft drink advertisements were present) was marginally associated with lower mean daily participation in the school lunch program (*p* < 0.07) [65], perhaps because such advertising increases students’ interest in purchasing *a la carte* items and they therefore opted out of the meal program. One Canadian study [61] found that neither the perceived presence of snack logos nor the perceived presence of beverage logos was significantly associated with self-reported overweight/obesity (*p* = 0.274 and *p* = 0.812, respectively).

## 4. Discussion

This review offers a broad description of the most common approaches and measures used to examine the school-based food and beverage marketing environment. The majority of studies used direct observations, self-report or in-person/telephone interviews, and assessed some combination of direct advertising, indirect advertising and product sales. The number and types of marketing features measured was highly variable across studies making direct comparisons between studies or geographic regions challenging. There is currently no “gold standard” instrument or measures of school food and beverage marketing exposure and little psychometric testing or validation of tools has been reported to date. Where appropriate, intra- or inter-rater agreement and test-retest reliability should be conducted to give future users an idea of how reproducible findings may be. To assess validity, comparing multiple instruments (e.g., self-report with direct observation) would be important in determining instrument quality and convergence. Moreover, no studies addressed issues pertaining to participants’ ability to fully report on all aspects of the food and beverage marketing environment in schools. As such, we do not know yet how comprehensive, accurate, or biased subjective reports might be and how they compare with other more objective assessment tools. Future research could address these issues or attempt to measure potential biases. 

This review also offers a summary of the degree to which students are exposed to food and beverage marketing and, a preliminary understanding of whether exposure differs by student- and/or school-level characteristics. As noted elsewhere [34], this review confirms that exposure to school food and beverage marketing is common in the US, but varies depending on geographic context. Emerging evidence from other countries indicates similar results, yet research with larger, nationally representative samples are needed to generalize such findings in other jurisdictions such as Canada where there is growing federal discussion of restricting commercial marketing of unhealthy foods and beverage to children [37]. Moreover, because studies have tended to focus on just some marketing elements and are therefore not sufficiently comprehensive, it is difficult to understand the totality of food and beverage marketing that students are exposed to. Developing a core set of measures that can be used by researchers, policy-makers, schools or community groups may be particularly useful in helping key stakeholders track changes over time and make comparisons across samples. Moreover, standardized measures could help determine how much exposure is “a lot” and ultimately meaningful to inform and support targeted policy efforts designed to restrict unhealthy food and beverage marketing and shift students’ food and beverage choices to products that meet national or international nutritional guidelines. 

Evidence suggests that the overall prevalence of advertising was significantly higher among secondary schools than middle/elementary schools, likely owing to differences in food environment characteristics and policies between these contexts. For instance, North American secondary schools generally offer students more opportunities to purchase food on campus than middle/elementary schools [70], making these settings more attractive to food and beverage companies that wish to promote their products and harness the purchasing power of students. Additionally, some work pointed to differences in the prevalence of school food and beverage marketing by SES, echoing work conducted on television suggesting lower SES groups are exposed to greater amounts of advertising [31]. These findings may be the result of lower-income schools attempting to generate funds to fill budgeting gaps [30,71]. Regardless, slightly more than half of the studies examined differences in exposure to advertising by student- and/or school-level characteristics, and more work, particularly with larger representative samples, is needed to conclude that systematic differences in exposure to food and beverage marketing exist in schools. 

Findings from this review demonstrate that at school, much of the food and beverage marketing children see is for items they are discouraged from consuming by national dietary guidelines and nutrition education messaging. However, studies measuring the relative healthfulness of marketed items used different regional or national nutritional metrics and definitions of healthy foods. Future research should consider using standardized measures, either national or international, that quantify the extent to which the food and beverage products marketed to children and adolescents are high in sugar, sodium, and saturated fat. Doing so would allow for comparisons across school-based studies, as well as to those set in other child-oriented locations (e.g., daycares, recreation centers). Few studies have assessed whether exposure to marketing is associated with students’ dietary intake at school and if so, which types, quantities and duration of exposure are most salient. Still, preliminary evidence suggests that exposure to school food and beverage marketing may shape children’s food purchasing and consumption, particularly for minimally nutritious food items. Given the cross-sectional nature of these studies, it is also possible that youth who purchase and consume such items may be more likely to pay attention to marketing practices. Comprehensively measuring the full spectrum of marketing activities within schools may contribute to improved understanding of such an ‘exposure’ threshold, and whether one form of marketing is more or less important than others in influencing food practices. Future research should move beyond focusing solely on describing the school food and beverage marketing environment and consider testing whether exposure is associated with meaningful diet-related outcomes among students, for example cognitive (e.g., brand awareness/recognition), behavioral (e.g., food purchases, dietary intake), or health-related (e.g., weight perceptions and chronic disease risk factors). Obtaining qualitative insight regarding perspectives of students, parents, and school stakeholders may also prove useful. Moreover, longitudinal monitoring is needed for determining if emerging policies aimed at reducing exposure impact what children see and ultimately choose to eat.

To our knowledge, this study is the first comprehensive review focusing on food and beverage marketing in schools. Offering a narrative integration of the relevant evidence illustrates important gaps in knowledge and measurement, which can inform and advance school food initiatives. Articles were gathered from a variety of databases and grey literature sources, aiding in the comprehensiveness of the chosen studies. We retained all references with a broad focus on the school food environment in our initial round of review, before looking more closely at whether aspects of food and beverage marketing were present. Despite these efforts, it is possible that we missed other measures embedded in larger school food environment studies or unpublished evaluations. Nevertheless, this review is timely given recent policy recommendations put forth by non-governmental organizations urging government entities to protect children from influential food and beverage marketing practices [38,72]. 

## 5. Conclusions

This review indicates that the evidence base on this topic, while growing, is still nascent both in terms of the number and comprehensiveness of studies. Children are exposed to a significant amount of unhealthy food and beverage marketing at schools in the US, and to some extent in Canada and Europe. Yet, research must determine whether (and if so, how much) exposure is important in influencing students’ diet-related outcomes while at school, using sampling approaches that allow findings to be generalized to locations outside of the US. To do this, a core set of standard and universal measures are needed that are sufficiently rigorous and comprehensive to assess the totality of school food and beverage marketing practices that students are exposed to. Agreeing on such measures is challenging, however, a standard set of measures (that can be expanded upon based on need) could prove useful in comparing exposures (and related outcomes) in diverse populations, and for identifying effective strategies to mitigate the potentially harmful impacts of food and beverage marketing on children. 

## Supplementary Materials

The following are available online at www.mdpi.com/1660-4601/14/9/1054/s1, Table S1: Approaches used to measure the food and beverage marketing environment in elementary, middle, and secondary schools (*n* = 27 studies) ^a^, Table S2: Current evidence of school food and beverage marketing exposure, differences in exposure by student- or school-level characteristics, and associations with children’s diet-related outcomes among examined studies (*n* = 27 studies), Search Strategy S1: Web of Science Core Collection, Search Strategy.
