# Supplementary material for: Food and Beverage Marketing in Schools: A Review of the Evidence
Source: Int J Environ Res Public Health. 2017 Sep 12;14(9):1054. doi: 10.3390/ijerph14091054 (PMC5615591; doi:10.3390/ijerph14091054)
Supplement: Supplementary file 1 [file ijerph-14-01054-s001.zip › IJERPH_SearchStrategy_FINAL_Velazquez et al.docx]

**[Web of Science Core Collection](javascript:;), Search Strategy**

| # 1. | TOPIC: ("data collection" or "focus group*" or interview* or questionnaire* or survey*) OR TOPIC: (assess* or tool* or instrument* or self-report*) OR TOPIC: (inventor* or checklist* or audit* or observation*) |
| --- | --- |
| # 2. | TOPIC: ((marketing OR advertis* or promotion* or sign or signs or poster* or logo* or "premium offer*") near/1 (food* OR beverage*)) OR TOPIC: ("nutrition environment*" or "food environment*" or "school food") |
| # 3. | TOPIC: (("fast food*" or "sugar sweetened beverage*" or "sugar-sweetened beverage*" or "energy drink*" or snack* or "soft drink*" or pop or soda* or juice* or "potato chip*" or chips or cand* or chocolate* or fruit* or vegetable* or low-nutrient or energy-dense or "a la carte" or pizza or hamburger* or cheeseburger* or ice cream or French fries or fries OR "sports drink*" OR "pouring right*")) |
| # 4. | TOPIC: (Adolescent* OR child* OR teen* OR youth OR "young people*" OR prepubescent OR pubescent) |
| # 5. | TOPIC: (School OR "primary school*" OR "elementary school*" OR "middle school*" or "secondary school*" OR "high school*") |
| # 6. | #5 OR #4 |
| # 7. | #3 AND #2 AND #1 |
| # 8. | #7 AND #6 |
| # 9. | (#7 AND #6) AND LANGUAGE: (English) |
| # 10. | (#7 AND #6) AND LANGUAGE: (English) AND DOCUMENT TYPES: (Article) |
