# Supplementary material for: Food and Beverage Marketing in Schools: A Review of the Evidence
Source: Int J Environ Res Public Health. 2017 Sep 12;14(9):1054. doi: 10.3390/ijerph14091054 (PMC5615591; doi:10.3390/ijerph14091054)
Supplement: Supplementary file 1 [file ijerph-14-01054-s001.zip › IJERPH_Table1_FINAL_Velazquez et al.docx]

**Table S1.** Approaches used to measure the food and beverage marketing environment in elementary, middle, and secondary schools (n=27 studies) among studies published between 2002-2015.^a^

| **Authors** | **Sample**  **Characteristics^b^** | **Marketing-Related Focus^c^** | **Data Collection Approach** | **Time to Administer** | **Instrument Development** | **Psychometric Properties** | **Examples of Marketing**  **Variables Measured** |
| --- | --- | --- | --- | --- | --- | --- | --- |
| Adachi-Mejia et al., 2013 | 26 schools that included grades 9-12, New Hampshire and Vermont, USA^ᴪ^ | Assessed how vending machine-front content was advertised in schools | Direct observations of schools conducted by trained coders | Not mentioned | Not mentioned | Not mentioned | Presence of beverage vending machine-front advertising, indicated by listing brand names shown (e.g., Dasani, Coca-Cola) |
| Briefel et al., 2009^1^ | 287 public elementary, middle and high school principals or food service directors; 2,314 students in grades 1-12, USA^b,ᴧ^ | Determined the presence of exclusive beverage contracts in schools; examined associations between exclusive beverage contracts and dietary intake | Self-report survey completed by principals or FSD (marketing) and students (diet) | Not mentioned | Not mentioned | Not mentioned | Presence of exclusive beverage contract with soft drink bottler (Yes/No) |
| Caspi et al., 2015^d,2^ | 505 middle and high school principals, Minnesota, USA^b,ᴪ^ | Examined school policies related to advertising LNED foods | Self-report survey completed by principals | Not mentioned | Not mentioned | Not mentioned | Restrict advertisements for candy, fast- food restaurants, or soft drinks in the following locations: 1) in school building; 2) on school grounds; 3) on school buses; and 4) in school publications (Yes/No) |
| Center for Science in the Public Interest, 2008 | 36 public elementary, middle, and high schools, Montgomery County, Maryland, USA^b,*^ | Assessed the nature and extent of food marketing in schools^c^ | Direct observations of schools completed by school nurses; phone interviews with principals conducted by dietetic interns | Observations took 2-3 hours per school; interviews took ~40 minutes | Instrument- and protocol- training; tool adapted from the CPL, FBMS | Not mentioned | Direct advertising (e.g., number of posters in cafeteria); indirect marketing including branded curricula (Yes/No); product sales including fundraisers (Yes/No); policies regarding food marketing (Yes/No) |
| Craypo et al., 2006 | 20 public high schools, grades 9-12, California, USA^ᴪ^ | Inventoried and assessed food and beverage marketing on school campuses^c^ | Direct observations of schools and in-person interviews with a principal completed by coordinator and 1-2 high school students | Observations took ~3 hours per school, including the 10-15 minute interview | Original CPL, FBMS Tool | Not mentioned | Direct advertising (e.g., number of posters in cafeteria); indirect marketing including branded curricula (Yes/No); product sales including fundraisers (Yes/No); market research (Yes/No); policies regarding food marketing (Yes/No) |
| Findholt et al., 2011 | 8 elementary schools, grade K-6, Union County, Oregon, USA^*^ | Assessed food and beverage messaging in schools | Direct observations of schools completed by college students | Not mentioned | Instrument training and field training, tool adapted from the CPL, FBMS | Intra- and inter-rater reliability; data collection began when reliability was at least 80% agreement | Total number, location (e.g., classroom, hallway), and type (e.g., healthy, unhealthy) of food messages |
| Finkelstein et al., 2008^1^ | 395 public elementary, middle, and high school principals, USA^b,ᴧ^ | Described school policies related to exclusive beverage contracts | Self-report surveys completed by principal and FSD | Not mentioned | Not mentioned | Not mentioned | Presence of exclusive beverage contract with soft drink bottler (Yes/No) |
| French et al., 2002 | 336 high school (grades 9-12) principals, Minnesota, USA^b,ᴪ^ | Described school practices related to beverage contracts, advertising and coupons | Self-report surveys completed by principal or assistant principal | Not mentioned | Developed based on literature, school nutrition guidelines, and researcher experience | Not mentioned | Presence of beverage contract with soft drink bottler (Yes/No); advertising allowed inside/outside cafeteria (Yes/No); distribution of coupons from outside companies allowed (Yes/No) |
| French et al., 2003 | 18 principals and 19 FSD from 20 high schools (grades 9-12) Minnesota, USA^*^ | Described school practices related to beverage contracts, advertising, and coupons | Self-report surveys completed by principal and FSD | Not mentioned | Developed based on previously published surveys | Not mentioned | Presence of school/district soft drink contract (Yes/No); advertising allowed inside/outside cafeteria (Yes/No); distribution of coupons from food service/outside companies allowed (Yes/No) |
| Johnston et al., 2007^3,4^ | 345 public and private school principals; 37,543 students grades 8, 10, and 12, USA^b,ᴧ^ | Examined school policies related to beverage contracts, and advertising | Self-report surveys completed by administrators (marketing) and students (demographics) | Not mentioned | Not mentioned | Not mentioned | Presence of a beverage contract with soft drink bottler (Yes/No); whether the soft drink was advertised on school grounds or during school events (Yes/No); the types of advertising and promotions allowed (e.g., posters, coupons) (Yes/No) |
| Johnston et al., 2015^e,5^ | 612 middle and high school principals, USA^b,ᴧ^ | Explored in-school food marketing policies, including exclusive beverage contracts | Self-report surveys completed by principal | Not mentioned | Not mentioned | Not mentioned | Presence of school/district exclusive beverage contract (Yes/No); sales incentives (Yes/No); and types of advertising and promotions allowed (e.g., posters, coupons) (Yes/No) |
| Kelly et al., 2010 | 331 post-primary (e.g., secondary, vocational, or community) school principals or staff, Republic of Ireland^b,ᴧ^ | Investigated food-based commercial activity in schools^c^ | Self-report survey completed by principals or staff | Not mentioned | Pilot tested | Not mentioned | Accepts sponsorship from for-profit organisations (Yes/No); type of sponsorship activity allowed (e.g., equipment, curricula) (Yes/No) |
| Larson et al., 2014^f,2^ | 261 middle/high school principals, Minnesota, USA^b,ᴪ^ | Examined school policies related to food advertising; explored associations between advertising and dietary intake | Self-report survey completed by principals (marketing) and students (diet, (demographics) | Not mentioned | Not mentioned | Not mentioned | Ban advertisements for candy, fast-food restaurants, or soft drinks in the following locations: 1) in school building; 2) on school grounds; 3) on school buses; and 4) in school publications (Yes/No) |
| Latimer, 2013 | 30 middle schools, grades 6-8, Austin, Texas, USA^*^ | Documented food and beverage promotions (e.g., posters, product sales) in schools^c^ | Direct observations of schools completed by project staff (one researcher, one trained assistant) | Observations took ~ 1 hour per school | Pilot testing; field training | Inter-rater reliability (average % agreement) of coding tool ranged from 92-97% | Number and type of food and beverage promotions; product description, location, and relative healthfulness |
| Mazur et al., 2008 | 44 primary and secondary schools, Rzeszow, Poland^*^ | Assessed foods advertised in schools; examined associations between food advertising and children’s food purchasing habits^c^ | Direct observations of schools completed by research team; self-report surveys completed by  principals (marketing) and store owners (food purchases) | Not mentioned | Not mentioned | Not mentioned | Number and type of food advertisements in school store windows; presence of direct corporate advertising in proximity of the stores or in school buildings, classrooms, corridors, or gymnasiums (Yes/No); relative healthfulness of foods advertised; schools’ policies about advertising and food company sponsorship |
| McDonnell et al., 2006 | 228 high school (grade 9-12) FSD and 79 principals, Pennsylvania, USA^b,ᴪ^ | Described perceptions of the extent and enforcement of school policies related to food advertisements | Self-report survey completed by FSD and principals | Not mentioned | Pilot testing | Telephone interviews with ~15% of respondents to validate survey results | Restrict food advertisements on school grounds (Yes/No) |
| Minaker et al., 2011 | 4,936 students from 136 secondary schools (grades 7-10), Alberta, Canada^ᴪ^ | Examined students’ perceived presence of food/beverage logos at school; explored associations between presence of logos and diet-related outcomes and overweight/obese | Self-report web-based survey completed by students | ~45 minutes | Not mentioned | Not mentioned | Perceived presence of snack (e.g., chip, candy or chocolate bar company) and beverage (e.g., soft-drink company) logos (Yes/No) in school |
| Molnar et al., 2008 | 391 primary (elementary and middle) and high school principals or FSD, USA^b,ᴧ^ | Measured the nature and extent of food marketing activities in schools^c^ | Telephone survey with school principals or FSD | Not mentioned | Not mentioned | Not mentioned | Engagement in fundraising, incentive programs, school programs or activities, exclusive marketing agreements, electronic marketing, appropriation of space, and/or sponsorship of supplementary educational materials with companies selling FHFS and/or FMNV (Yes/No) |
| Nanney et al., 2013^6^ | 6,732 middle and junior/high school (grade 6-12) principals, USA^b,ᴧ^ | Examined school policies related to food marketing | Self-report survey completed by principals | Not mentioned | Not mentioned | Not mentioned | Ban advertisements for candy, fast-food restaurants, or soft drinks in the following locations: 1) in school building; 2) on school grounds; 3) on school buses; and 4) in school publications (Yes/No) |
| Phillips et al., 2010^g^ | 832 elementary, middle and high school principals, Arkansas, USA^ᴪ^ | Assessed school policies related to food advertising and coupons | Self-report survey completed by principals | Not mentioned | Items drawn from national sources (e.g., SHPPS) when possible | Not mentioned | Restrict commercial advertising by food/beverage companies on school campuses (Yes/No); restrict use of food or food coupons as reward in classrooms (Yes/No) |
| Polacsek et al., 2012 | 20 urban and rural high schools, Maine, USA^b,ᴪ^ | Assessed the nature and extent of junk food marketing, including policies, practices, and compliance with legislation in schools^c^ | Direct observations of schools and in-person interviews with principal or FSD completed by research assistants | Observations took ~2 hours per school | Field training; tool adapted from the CPL, FBMS | Inter-rater reliability using Spearman correlations (ranged from 0.56-0.72); intra-class correlation (not consistently strong – used records from one researcher) | Location of food marketing, number of posters/signs and/or product logos, product name or logo, compliance with statewide marketing ban (Yes/No); presence of marketing related to media, equipment and supplies, and activities, sponsorships, scholarships, and fundraising (Yes/No) |
| Probart et al., 2006 (a) | 228 high school (grades 9-12) FSD, Pennsylvania, USA^b,ᴪ^ | Assessed soft drink advertising in schools; examined associations between soft drink advertising and school lunch participation | Self-report survey completed by FSD | Not mentioned | Pilot testing | Telephone interviews with ~15% of respondents to validate key variables; validity determined by triangulation of 3 questions from survey and telephone interviews | Number of locations where soft drink advertisements exist within school (range 0-6 potential locations); presence of soft drink machines owned by a company and school/district receives incentives (Yes/No) |
| Probart et al., 2006 (b) | 228 high school (grades 9-12) FSD, Pennsylvania, USA^b,ᴪ^ | Described the extent and location of soft drink advertisements in schools | Self-report survey completed by FSD | Not mentioned | Pilot testing | Phone interviews with ~15% of respondents to validate key variables; validity determined by triangulation of 3 questions from survey and telephone interviews | Number of locations where soft drink advertisements exist within schools (range 0-6 potential locations); incentives provided to school/district by food company (Yes/No); existence of exclusive beverage contract (Yes/No); subscription to Channel One (Yes/No) |
| Terry-McElrath et al., 2012^3^ | 757 middle and 762 high school principals, USA^b,ᴧ^ | Examined school policies related to exclusive beverage contracts | Self-report survey completed by school principals | Not mentioned | Pilot testing | Not assessed | Existence of exclusive beverage contract (Yes/No); cash or other incentives provided to school or district (Yes/No) |
| Terry-McElrath et al., 2014^h,3,7^ | 3,785 elementary, 1,594 middle, and 1,568 high school principals or FSD, USA^b^ | Examined food-based commercialism in schools^c^ | Self-report survey completed by school principals or FSD | Not mentioned | Pilot testing | Not assessed | Existence of exclusive beverage contract (Yes/No); incentives provided by beverage company (Yes/No); presence of posters/signs for soft drinks, fast food restaurant, or candy (Yes/No); use of other marketing (e.g., textbook covers, coupons, sponsorship of school events) (Yes/No) |
| Turner et al., 2012^i,5^ | 680 public and 313 private elementary school principals, USA^b,ᴧ^ | Examined school policies related to exclusive beverage contracts | Self-report surveys completed by school principals | Not mentioned | Cognitively tested with principals to ensure comprehension | Not mentioned | Existence of exclusive beverage contract (Yes/No) |
| Velazquez et al., 2015 | 23 elementary and secondary schools, Vancouver, British Columbia, Canada^*^ | Documented food-related advertising, messaging and signage in schools^c^ | Direct observations of schools completed by two researchers | Observations took ~1-2 hours per school | Tool adapted from other sources (e.g.., school- and television-based tools) | Inter-rater reliability of coding tool using Kappa coefficients (range 0.78-0.95) | Location, size, main purpose, category of food depicted (e.g., fruit and vegetables), and classification (e.g., choose most, choose sometimes) of foods advertised; presence of common marketing techniques (e.g., logos, animated characters) (Yes/No) |

Abbreviations: CPL, FBMS (California Project Lean, Food and Beverage Marketing in Schools Assessment Tool); FHFS (Foods High in Fat or Sugar); FMNV (Foods of Minimal Nutritional Value); FRPL (Free/Reduced Price Lunch); FSD (Food Service Director); LNED (Low-nutrient, energy-dense)

*= city/region-, ᴪ=state/province-, ᴧ= national-level sample of children, administrators, or schools

^a^ Some of the articles included in this review used other tools to examine measures and associations unrelated to our primary objectives. We do not report these here.

^b^ Randomly selected or census sample of schools, administrators, and/or students

^c^ Marketing was the primary focus of the study

^d^ Repeat cross-sectional study conducted with principals in 2008 (n=203), 2010 (n=226), and 2012 (n=275); 505 unique schools were included in the analysis; findings presented here are the average prevalence differences across all years.

^e^ Repeat cross-sectional study conducted with principals or food service directors annually across eight years (2007-2014); total n=4,983 for all years combined; findings presented here are for 2014 only.

^f^ Repeat cross-sectional study conducted with principals in 2008 (n=192), 2010 (n=261), and 2012 (n=261); findings presented here are for 2012 only.

^g^ Repeat cross-sectional study conducted with principals across five years (2004-2008); findings presented here are for 2008 only.

^h^ Repeat cross-sectional study conducted with principals annually across six years (2007-2012); total n=6,947 for all years combined; findings presented here are for 2012 only.

^I^ Repeat cross-sectional study conducted with principals in 2006/2007 and 2009/2010; findings presented here are from 2009/2010 only.

^1^ SNDA-III (Third School Nutrition and Dietary Assessment) study

^2^ MN-SHPPS (Minnesota School Health Profiles Principal Survey) study

^3^ YES (Youth, Education, and Society) study

^4^ MTF (Monitoring the Future) study

^5^ BTG (Bridging the Gap) study

^6^ SHPPS (School Health Policies and Practices Survey)

^7^ FF (Food and Fitness) study
