# Supplementary material for: Food and Beverage Marketing in Schools: A Review of the Evidence
Source: Int J Environ Res Public Health. 2017 Sep 12;14(9):1054. doi: 10.3390/ijerph14091054 (PMC5615591; doi:10.3390/ijerph14091054)
Supplement: Supplementary file 1 [file ijerph-14-01054-s001.zip › IJERPH_Table2_FINAL_Velazquez et al.docx]

**Table S2.** Current evidence of school food and beverage marketing exposure, differences in exposure by student- or school-level characteristics, and associations with children’s diet-related outcomes among examined studies (n=27 studies) published between 2002-2015.

| **Study** | **Student or School Characteristic Measures** | **Diet-Related Measures** | **Key Findings**  **Exposure to Marketing** | **Key Findings**  **Exposure to Marketing by Student- or School-Level Characteristics** | **Key Findings**  **Association between Exposure to Marketing and Diet-Related Outcomes** |
| --- | --- | --- | --- | --- | --- |
| Adachi-Mejia et al., 2013 | Geographic location (i.e., urban, rural, or town) | N/A | All schools had at least one vending machine with an advertisement; most (83.2%, n=94) machines featured advertisements. The most common advertisements were for branded bottled water (e.g., Dasani) (69.1%, n=65). Advertisements for SSBs (e.g., Snapple, Gatorade) were on almost one-third of vending machines (27.7%, n=26), whereas milk was advertised on 4.3% (n=4) of machines. | No statistically significant differences in advertising by geographic location. | N/A |
| Briefel et al., 2009 | School level (i.e., elementary, middle, and high school) | Energy from SSBs, LNED foods, and fruit and vegetables consumed at school | N/A | The proportion of elementary, middle, and high school students (43%, 35%, and 16%, respectively) attending schools without an exclusive beverage contract was significantly different (p=0.017). | Compared to schools with exclusive beverage contracts, energy from SSBs was estimated to be 16kcal/day lower (p<0.05) among students attending middle schools without an exclusive beverage contract; while energy from LNED items was 29kcal/day higher (p<0.05) among students attending elementary schools without an exclusive beverage contract. |
| Caspi et al., 2015 | School level (i.e., middle, junior/senior high and high schools); geographic location (i.e., city, suburb, town/rural); FRPL eligibility (i.e., <20%, 20%-<60%, ≥60%); minority enrollment (<5%, 5%-<50%, ≥50%) | N/A | Schools were significantly less likely to ban advertisements for LNED items on school grounds over time (2008-2012) (p=0.01). | City schools were significantly more likely to ban advertisements for LNED items in school buildings and on school grounds than rural/town schools; suburban schools were significantly more likely to ban advertisements for LNED items on school grounds than rural/town schools. | N/A |
| Center for Science in the Public Interest, 2008 | School level (i.e., (elementary, middle and high schools) | N/A | The majority (83%) of schools had posters with food marketing, most (58%) of which was for restaurants, prepared foods, and soft drinks. Four out of five vending machines had food marketing. Branded fundraisers and giveaways were common (50% and 25% of schools, respectively); marketing using school supplies (19%), curricula (11%), sponsorships (3%), or scholarships (0%) were less frequent. Less than half (47%) of schools reported having a food marketing policy. | The amount of food marketing varied by school level; 79% of elementary, 100% of middle, and 83% of high schools had food-related posters; the proportion of posters for healthier foods was highest in elementary (55%), compared with middle (40%) and high (18%) schools; elementary schools reported more frequent use of branded curricula or proof-of-purchase programs, whereas marketing on vending machines and through fundraisers was more prevalent in middle and high schools. | N/A |
| Craypo et al., 2006 | N/A | N/A | Most (90%) schools had posters marketing food; 49% of posters had a nutrition education message and 51% promoted food products or brands. Close to 65% of vending machine advertisements were for SSBs. All schools reported holding branded food-based fundraisers, and 26% of schools used branded curricula. Less than half (45%) of schools had a policy addressing marketing, none of which was specific to food. | N/A | N/A |
| Findholt et al., 2011 | N/A | N/A | In classrooms, 60% (n=176) of food-related messages were for unhealthy products, compared to 29% (n=85) that were healthy products. | N/A | N/A |
| Finklestein et al., 2008 | School level (e.g., elementary, middle, high), racial/ethnic minority enrolment (e.g., ≤10%, >10%-45%, >45%), household income (e.g., ≤30% FRPL, >30%-50% FRPL, >50% FRPL) | N/A | Nearly 55% of all schools had an exclusive beverage contract (46% elementary, 59% middle, and 78% high schools). | Middle/high schools (69%) were significantly more likely than elementary schools (46%) to have an exclusive beverage contract (p=0.002). No statistically significant differences in the presence of an exclusive beverage contract were found by minority enrolment or household income. | N/A |
| French et al., 2002 | N/A | N/A | Most (77%) principals reported their school/district had a contract with one or more soft drink companies. Food advertising was allowed in and outside of school cafeterias in 20% and 35% of schools, respectively. And, 21% and 22% of schools allowed food/beverage coupons to be distributed from food service and outside companies, respectively. | N/A | N/A |
| French et al., 2003 | N/A | N/A | Thirty-nine percent of principals and 41% of FSD allow food advertisements inside their cafeteria (33% and 39%, respectively, allow them outside the cafeteria). Twenty-eight percent of principals and 74% of FSD allow coupons from food service to be distributed (24% and 22%, respectively, allow them from outside companies). Most principals (71%) and FSD (79%) report their school/district has a soft drink contract. | N/A | N/A |
| Johnston et al., 2007 | Student grade (i.e., middle, high school), racial/ethnic background (i.e., white, black, Hispanic or other), and SES (indicated by parental education, an average of paternal and maternal educational attainment). | N/A | N/A | A greater percentage of high school students (83%) compared with middle school students (67%) attended schools with a soft drink contract (p<0.01); more high school students (21%) than middle school students (7%) attended schools with soft drink advertisements (p<0.001); students in low-SES middle (8%) and high (29%) schools were exposed to more soft drink advertising than students in high-SES middle (4%) and high (13%) schools (p<0.05 and p<0.001, respectively); the percentage of students attending low-SES middle schools with sponsorship of school events was greater (23%) than students in high-SES middle schools (10%) (p<0.05). | N/A |
| Johnston et al., 2015 | Student grade (i.e., middle, high school) | N/A | N/A | Significantly more high school students (63%) than middle school students (47%) attend schools with an exclusive beverage contract (p<0.001); a greater percentage of high school students (49%) compared to middle school students (32%) are in schools that receive a sales percentage from exclusive contracts (p<0.001); more high school students (25%) than middle school students (14%) attend schools where sponsorship from food/beverage companies was present. The percentage of students in schools exposed to promotions for coupons, textbook covers, and posters was relatively small (≤10% for each). | N/A |
| Kelly et al., 2010 | N/A | N/A | Nearly 40% of schools accepted corporate sponsorship; sponsorship was prevalent on sports (71% of schools) and IT (48% of schools) equipment. Sponsorship of competitions (40%), educational materials (20%), vending machines (13%), and science equipment (9%) were less prevalent. | N/A | N/A |
| Larson et al., 2014 | School level (i.e., middle, high school); geographic location (i.e., city, suburban, town/rural); minority enrolment (i.e., <20%, ≥20%); FRPL eligibility (i.e., <40%, ≥40%) | School-level mean intakes of fruit and vegetables, as well as SSBs | Schools banned advertising for candy, fast-food restaurants, or soft drinks in a mean of 2.4±0.2 (out of 4) locations; the proportion of schools with policies that banned advertising by location was 46% for school grounds, 57% for publications, 61% for school buildings, and 70% for buses. | No statistically significant differences in the mean number of locations where schools banned advertising by school characteristics. | The total number of locations where a school banned advertising in any of the 4 specific locations assessed were unrelated to school-level means for students’ dietary behaviors. |
| Latimer, 2013 | FRPL eligibility (i.e., <60%, ≥60%) as a proxy for economic disadvantage; minority enrolment (i.e., <75%, ≥75%) | N/A | The most common type of advertisement was for nutrition education (30%); the majority (58%) of advertisements were in cafeterias; a slightly higher proportion of unhealthy (51%) compared to healthy (49%) advertisements was found. | The mean number of advertisements was higher for more affluent schools (87, SD=30) than more economically disadvantaged schools (73, SD=28) (p<0.001); the mean number of advertisements was higher for schools with a lower percent minority (93, SD=29) than schools with a higher percent minority (60, SD=17) (p<0.001). | N/A |
| Mazur et al., 2008 | School level (i.e., primary, secondary) | Food purchased from school stores | Around 40% of store windows advertised ‘healthy’ foods; 9% of stores displayed advertisements for food companies, none of which were for healthy foods. | No statistically significant differences in advertising by school type. | Advertising a speciﬁc food was associated with purchase of that food (p<0.001). |
| McDonnell et al., 2006 | N/A | N/A | Approximately 20% of principals and FSD report that they do not have a policy or recommendation restricting food advertisements on campus. | N/A | N/A |
| Minaker et al., 2011 | N/A | Frequency of purchasing food and/or beverages from vending machines; frequency of consuming salty snacks, candy and soft drinks; overweight/obese | Approximately 40% of students reported the presence of snack logos in their schools. Whereas slightly over half (57%) of students reported the presence of beverage logos in their schools. | N/A | Perceived presence of snack or beverage logos was significantly associated with higher frequency of purchasing food and/or beverages from vending machines; students who reported the presence of snack logos consumed salty snack and candy more frequently than students who reported no logos (p<0.0001 for both); the reported presence of beverage logos was not associated with frequency of soft drink consumption; perceived presence of snack or beverage logos was not associated with being overweight/obese. |
| Molnar et al., 2008 | N/A | N/A | Corporate-sponsored fundraising programs were the most frequently cited marketing activity (36% of all schools; 38% of primary schools), followed by incentive programs (26% all; 32% primary) and exclusive agreements (21% all; 16% primary). Sponsorship of program or activities (11% of all schools), appropriation of space (7% of all schools), and sponsorship of supplementary educational materials (2% of all schools) were least frequently reported. | N/A | N/A |
| Nanney et al., 2013 | Geographic location (i.e., town/rural, urban, suburban), minority enrolment (i.e., <5%, 5%-<50%, ≥50%), FRPL eligibility (i.e., <20%, 20%-<60%, ≥60%) | N/A | On average, schools banned marketing in 2.6±1.64 locations (out of 4). | The mean number of locations where advertising was banned was significantly higher (i.e., better) in urban and suburban schools than town/rural schools; the mean number of locations where advertising was banned was significantly higher in schools with a greater percentage of minority students than schools with low/medium minority enrolment. | N/A |
| Phillips et al., 2010 | School level (i.e., elementary, middle, and high school) | N/A | Forty-three percent of schools reported a policy prohibiting commercial food advertising by food companies on campus; 57% of schools indicated having a policy prohibiting use of food or food coupons as a reward in classrooms. | The presence of policies varied by school level; 48%, 46% and 28% of elementary, middle, and high schools, respectively reported a policy prohibiting commercial food advertising on campus; 61%, 56% and 49% of elementary, middle, and high schools had a policy prohibiting the use of food or food coupons as a reward in classrooms.^1^ | N/A |
| Polacsek et al., 2012 | N/A | N/A | Schools had an average of 49 food or beverage posters/signs (including vending machine facades); most posters/signs were in the cafeteria (52%); the most frequently marketed products were Dasani (10%), Coke (9%), Gatorade (8%), Aquafina (7%), and Vitamin Water (5%); products owned by Coca-Cola or Pepsi comprised 45% of all marketing instances; marketing via fundraising, sponsorship of events, and scholarships was present in 70%, 45%, and 20% of schools, respectively; marketing of non-compliant products was found in 85% of schools, with an average of 12 instances per school. | N/A | N/A |
| Probart et al., 2006 (a) | N/A | A la carte food sales; and mean daily participation in school lunch program | Soft drink advertisements exist in schools in an average of 1.1±1.0 locations; 63% of respondents reported the soft drink machines in their schools were owned by a soft drink company and school/district receives incentives. | N/A | Schools with a higher number of locations where soft drink advertisements were present had lower mean daily participation in the school lunch program (p=0.07). |
| Probart et al., 2006 (b) | N/A | N/A | Nearly two-thirds (63%) of respondents reported existence of soft drink machines owned by a company; 37% received incentives (e.g., money) based on sales; and 49% had an exclusive beverage contract. Soft drink advertisements were present in at least one location for approximately 65% of schools; advertisements were most prevalent on vending machines (62% of schools), followed by school grounds (e.g., playing fields) (27% of schools), school cafeterias (11% of schools), and other areas of the school (9% of schools). | N/A | N/A |
| Terry-McElrath et al., 2012 | School level (i.e., middle, high school) | N/A | N/A | Significantly more high school (75%) than middle school (65%) students attended schools with exclusive beverage contract; for schools with a beverage contract, about 50% of middle and high school students attended schools that receive either a portion of contract incentives or sales receipts. | N/A |
| Terry-McElrath et al., 2014 | Student grade (i.e., elementary, middle, high); race/ethnicity (i.e., White, Black, Hispanic, other); and FRPL eligibility (i.e., <15%, 15%-39%, ≥40%) as a proxy for SES | N/A | Approximately 3%, 50%, and 70% of students attended elementary, middle and high schools with exclusive beverage contracts; elementary students were frequently exposed to food coupons (64% of students), followed by posters or other displayed advertisements (6% of students); secondary school students were frequently exposed to event sponsorships (10% middle school, 21% high school), as well as coupons (6% middle, 5% high), and poster or other displayed advertisements (2% middle and high). | The percentage of students attending schools with beverage supplier advertising, text book cover advertisements, and school event sponsorship was significantly higher in high schools than middle schools (p<0.01 for each); differences in exclusive beverage contracts by student race/ethnicity were observed at the both the middle and high school levels (where exposure was significantly higher for students attending predominately white versus black schools); students attending predominately white versus black or Hispanic schools were significantly more likely to be exposed to school event sponsorships; middle and high school students attending high-SES schools were significantly less likely than students in mid- or low-SES schools to have an exclusive beverage contract. | N/A |
| Turner et al., 2012 | School type (i.e., public, private) | N/A | N/A | Significantly more public (93%) than private (84%) schools were without an exclusive beverage contract (p=0.023). | N/A |
| Velazquez et al., 2015 | School level (i.e., (elementary and secondary) | N/A | A total of 493 food or beverage promotions were identified; all secondary schools and 80% of elementary schools contained promotions (median=17, range=0,57); around 25% of promotions were for “choose least” or “not recommended” items, per provincial nutrition guidelines; nearly 1/3 of promotions were for branded items; only 13% conveyed nutrition education messages. | Promotions were significantly more common in secondary schools than elementary schools (p<0.01). | N/A |

Abbreviations: FRPL (Free/Reduced Price Lunch); FSD (Food Service Director); IT (Information Technology); LNED (Low-Nutrient, Energy Dense); SSBs (Sugar-Sweetened Beverages; SES (Socio-Economic Status)

^1^ These findings were descriptive in nature, and did not formally test whether exposure to food and beverage marketing differed by school-characteristics.
